# High-fat diet feeding significantly attenuates anagliptin-induced regeneration of islets of Langerhans in streptozotocin-induced diabetic mice

**DOI:** 10.1186/s13098-015-0047-y

**Published:** 2015-06-02

**Authors:** Takanori Shinjo, Yusuke Nakatsu, Misaki Iwashita, Tomomi Sano, Hideyuki Sakoda, Hisamitsu Ishihara, Akifumi Kushiyama, Midori Fujishiro, Fusanori Nishimura, Tomoichiro Asano

**Affiliations:** Section of Periodontology, Kyushu University Faculty of Dental Science, 3-1-1 Maidashi, Fukuoka, Higashi-ku Japan; Division of Molecular Medical Science, Department of Medical Chemistry, Graduate School of Biomedical Sciences, Hiroshima University, 1-2-3 Kasumi, Hiroshima, Minami-ku 734-8551 Japan; Division of Cervico-Gnathostomatology, Department of Dental Science for Health Promotion, Graduate School of Biomedical Sciences, Hiroshima University, Hiroshima, Japan; Department of Internal Medicine, Graduate School of Medicine, University of Tokyo, 7-3-1 Hongo, Tokyo, Bunkyo-ku Japan; Division of Diabetes and Metabolic Diseases, Nihon University School of Medicine, 30-1 Oyaguchikami-machi, Tokyo, Itabashi-ku Japan; Division of Diabetes and Metabolism, Institute for Adult Disease, Asahi Life Foundation, 1-6-1 Marunouchi, Tokyo, Chiyoda-ku Japan

**Keywords:** DPP-4 inhibitor, Anagliptin, Streptozotocin, High-fat diet, Islet of Langerhans

## Abstract

**Background:**

DPP-4 inhibitors reportedly exert effects on both alpha and beta cells, and promote the proliferation and survival of beta cells. We investigated the effects of anagliptin on structurally-impaired islets of Langerhans in streptozotocin (STZ)-treated mice, fed either a normal or a high-fat diet. Pdx-1 expression in the pancreas and serum insulin/glucagon concentrations were also examined.

**Findings:**

Anagliptin treatment significantly up-regulated pancreatic Pdx-1 expression, with elevated serum glucagon-like peptide-1 concentrations, regardless of whether the diet was normal or high-fat. However, interestingly, the beta cell regeneration, structural normalization of islets of Langerhans including alpha cell: beta cell area ratios, and serum insulin elevation, all observed with anagliptin administration in the animals fed a normal diet, were markedly suppressed in the high-fat fed group.

**Conclusions:**

High-fat diet feeding clearly weakened the regenerative effects of anagliptin on the islets of Langerhans in STZ-treated mice. Our findings suggest the importance of normalizing lipid metabolism for full manifestation of DPP-4 inhibitor effects on the islets of Langerhans.

**Electronic supplementary material:**

The online version of this article (doi:10.1186/s13098-015-0047-y) contains supplementary material, which is available to authorized users.

## Background

Dipeptidyl peptidase 4 (DPP-4) inhibitors were developed to enhance glucose-induced insulin secretion by prolonging the activities of incretins such as gastric inhibitory polypeptide (GIP) and glucagon-like peptide-1 (GLP-1). Many reports have also presented data suggesting that DPP-4 inhibitors induce beta cell proliferation and promote survival, while suppressing glucagon secretion [1–3]. However, it is unclear whether or not the proliferative effect of DPP-4 inhibitors on beta cells observed in rodent models is also significant in human diabetic subjects.

In this study, first, using streptozotocin (STZ)-treated mice we showed that anagliptin induced regeneration of beta cells and structural recovery of pancreatic islets of Langerhans. Then, we examined whether or not the effects of anagliptin are exerted regardless of whether the diet is high-fat (HFD) or normal.

## Methods

### Materials

Anagliptin was provided by Sanwa Kagaku Kenkyusho Co., Ltd. The antibodies against insulin, glucagon, Ki67 and Hoechst were purchased from Cell Signaling Technology (Beverly, MA, USA) and Abcam (Cambridge, UK). Anti-rabbit and anti-mouse horseradish peroxidase-conjugated antibodies were obtained from GE Healthcare (Buckinghamshire, UK). All other reagents were of analytical grade.

### Animals

C57BL/6J male mice obtained from The Jackson Laboratory (Bar Harbor, ME, USA) were housed under climate-controlled conditions with a 12:12-h light–dark cycle and were provided standard food or high-fat chow and water *ad libitum*. All protocols were approved by the Institutional Review Board of Hiroshima University.

### Creating and sustaining STZ-induced diabetes in mice

After a 16 h fast, 6-week-old C57BL/6J mice were injected with 200 mg/kg body weight STZ (Wako, Tokyo, Japan; freshly made in 0.1 M citrate buffer, pH 4.5) to induce severe diabetes. After a week, the mice with blood glucose levels exceeding 400 mg/dl were selected and divided into 4 groups (*n* = 6 each group), which were then fed normal chow (AIN-93 M, 76 % carbohydrate, 15 % protein and 9 % fat), normal chow premixed with 0.3 %(*w/w*) anagliptin (NA), high-fat chow (HFD-60, 7.5 % carbohydrate, 24.5 % protein and 60 % fat), or high-fat chow premixed with 0.3 %(*w/w*) anagliptin (HA) for 10 weeks. All chows were produced by Oriental Yeast Co., Ltd. (Tokyo, Japan). To prevent severe hyperglycemia caused by insulin deficiency, all mice were subcutaneously injected with Lantus® (Sanofi K.K., Tokyo, Japan) from 50 to 100 IU/g body weight, decided according to their blood glucose levels, once per day. All mice were sacrificed for subsequent analysis 24 h after the final Lantus® administration.

### Immunohistochemical analysis

Extirpated pancreases from the mice treated with STZ and anagliptin, fed the normal diet or the HFD, were fixed in 4 % paraformaldehyde for 48 h and subsequently embedded in paraffin. Pancreatic sections from mice given phosphate buffered saline alone served as controls. Sections were immune-labeled with rabbit anti-glucagon or anti-Ki67 followed by mouse anti-insulin. Digital images were captured with a fluorescence microscope BZ-9000 equipped with image analysis application (KEYENCE, Osaka, Japan). The insulin-positive beta cell: glucagon-positive cell area ratios were calculated by digitizing images captured through the 20-fold objective lens using ImageJ software. Images of 5 randomly chosen fields were captured from each pancreatic section.

### Measurement of mRNA expression by real-time PCR

Total RNA was isolated using Sepazol-RNA 1 (NakaLai Tesque, Kyoto, Japan), and 1 μg of RNA was reverse transcribed with Transcriptor Reverse Transcriptase (Roche Applied Science, Basel, Switzerland). The amplification reaction assay was performed using SYBR Premix Ex Taq (TaKaRa, Shiga, Japan) according to the manufacturer’s protocol. The primers were as follows: mouse forward pancreatic and duodenal homeobox 1 (Pdx-1) 5′-CATCTCCCCATACGAAGTGC-3′, mouse Pdx-1 reverse 5′-GGGGCCGGGAGATGTATTTG-3′; mouse musculoaponeurotic fibrosarcoma oncogene family proteins A (MafA) forward 5′-TTCAGCAAGGAGGAGGTCAT-3′, mouse MafA reverse 5′-CCGCCAACTTCTCGTATTTC-3′; mouse NeuroD forward 5′-CTTGGCCAAGAACTACATCTGG-3′, mouse NeuroD reverse 5′-GGAGTAGGGATGCACCGGGAA-3′; mouse NK6 homeodomain 1 (Nkx6.1) forward 5′-CTGCACAGTATGGCCGAGATG-3′, mouse Nkx6.1 reverse 5′-CCGGGTTATGTGAGCCCAA-3′; mouse GAPDH forward 5′-TGACGTGCCGCCTGGAGAAA-3′, mouse GAPDH reverse 5′-AGTGTAGCCCAAGATGCCCTTCAG-3′. Post-PCR melting curves confirmed the specificity of single-target amplification. Fold changes in the expressions of Pdx-1 relative to GAPDH were determined in triplicate.

### ELISA

Serum insulin, glucagon (Yanaihara Institute Inc., Shizuoka, Japan) and GLP-1 (Wako) concentrations were measured using ELISA kits according to the manufacturers’ instructions. Absorbance at 450 nm was determined using a microplate reader (Bio-Rad Laboratories, Hercules, CA, USA).

### Statistical analysis

Data are expressed as means ± SE. Statistical analyses were performed using Student’s *t*-test. Values of *P* < 0.05 were considered to indicate a statistically significant difference.

## Results

**Fig. 1:**
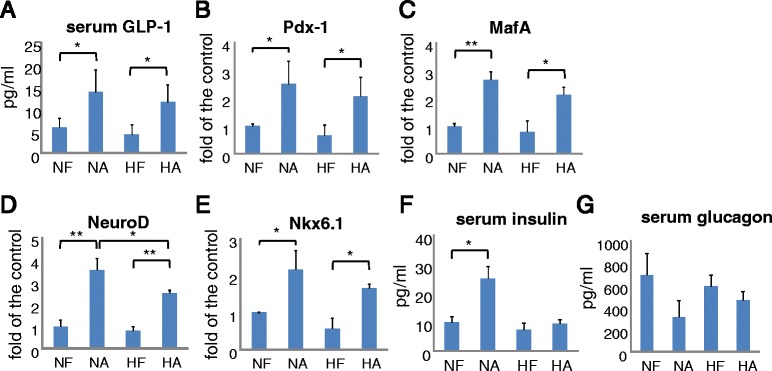
Effects of anagliptin on serum GLP-1, insulin, glucagon and beta-cell marker expressions in STZ-treated mice. **a** Serum GLP-1 levels in all mice were determined by ELISA. **b**–**e** The expressions of Pdx-1, MafA, NeuroD and Nkx6.1, beta cell marker genes, in each mouse pancreas were measured by real-time PCR. Data were calculated as values relative to the control (*NF*). **f** Serum insulin concentrations were measured by ELISA. **g** Serum glucagon concentrations were measured by ELISA. Quantitative data from 6 independent experiments are presented as *bar graphs*. *P < 0.05, Student’s *t*-test

**Fig. 2:**
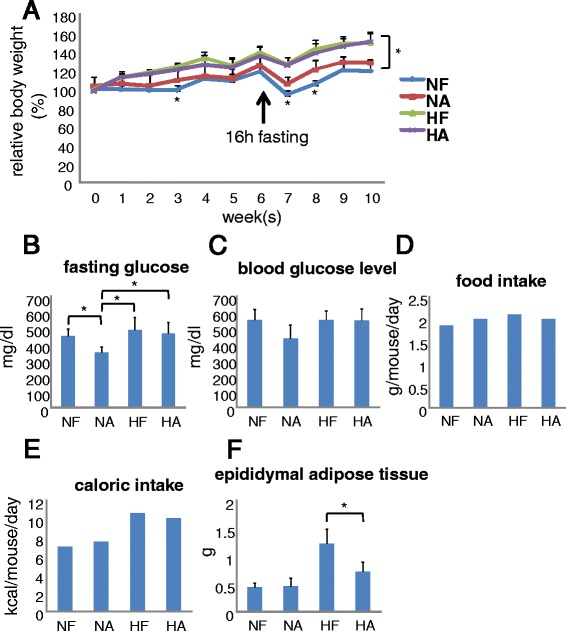
Characteristics of mice in each group. **a** Body weight change during the experiment in each group. At 6 weeks, mice were fasted for 16 h to determine fasting glucose levels. **b** Fasting glucose level after a 16 h fast. **c** Blood glucose level during final week. **d** Food intake of one mouse per day during the final week was calculated. **e** Caloric intake of one mouse per day was calculated by multiplying food intake and the caloric content of the each chow per gram (NF = 3802.7, HF = 5062 kcal/kg). **f** Epididymal adipose tissue weight in each group. Quantitative data from 6 independent experiments are presented as *bar graphs*. *P < 0.05, Student’s *t*-test

Serum GLP-1 concentrations rose with anagliptin treatment, with no difference being observed between the normal diet and HFD groups (Fig. 1a). Similarly, expression levels of Pdx-1, MafA, NeuroD and Nkx6.1, beta-cell markers reportedly associated with beta cell proliferation, differentiation, insulin production and homeostasis [4–6], were also up-regulated in the anagliptin-treated mice, regardless of whether the diet was normal or high-fat (Fig. 1b–e). On the other hand, serum insulin was significantly elevated by anagliptin in the normal diet fed mice but not in those receiving the HFD (Fig. 1f). Furthermore, serum glucagon was decreased by anagliptin in those given the normal diet, but the magnitude of this reduction was smaller in the HFD fed mice (Fig. 1g). HFD groups showed significantly increased body weight as compared with the normal diet groups (Fig. 2a). After a 16 h fast, among the 4 groups, NA had significantly lower plasma glucose (Fig. 2b). Blood glucose was lower, but not significantly, in NA than in the other three groups (Fig. 2c). Food intakes did not differ among the groups (Fig. 2d) but caloric intake was higher in the HFD than in the normal diet groups (Fig. 2e). Epididymal adipose tissue weight was markedly increased in HFD mice but anagliptin significantly blunted this elevation (Fig. 2f).

**Fig. 3:**
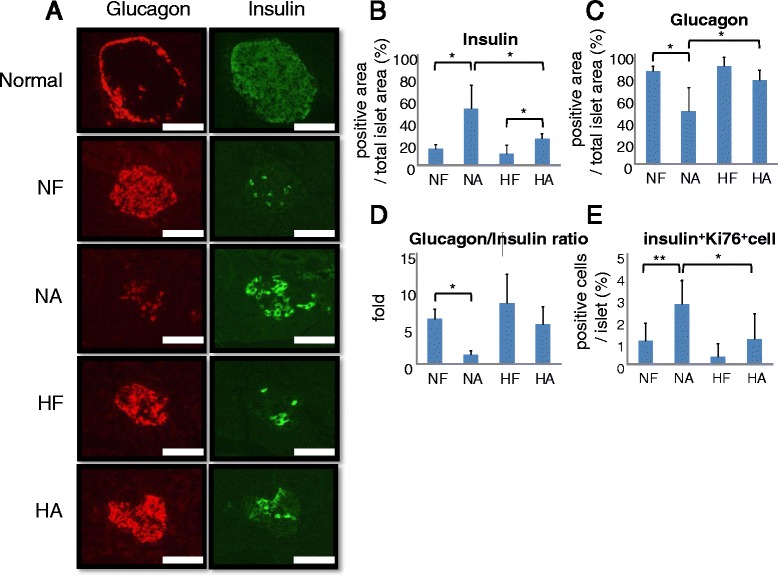
Effects of anagliptin on islet cell ratios and Ki67 expression in STZ-treated mice. **a** Islet immunohistochemical images stained with insulin or glucagon antibody in the normal controls and each of the STZ-treated groups of mice. *Scale bar* = 50 μm. **b**–**d** Insulin or glucagon positive area and glucagon/insulin positive area ratios (*lower*) of each STZ-treated group of mice were measured. **e** Islet immunohistochemical images stained with Ki67 or insulin antibody in each group. **f** Relative Ki67-insulin double positive cell area in islets from each STZ-treated mouse group. Quantitative data from 6 independent experiments are presented as *bar graphs*. *P < 0.05, Student’s *t*-test

Subsequently, islet insulin and glucagon positive areas were determined by immunostaining with anti-insulin and anti-glucagon antibodies, respectively (Fig. 3a). Anagliptin was found to markedly increase the insulin-positive cell area in the pancreases of STZ-treated mice fed a normal diet, while these increases were smaller in the HFD fed mice (Fig. 3b). On the other hand, only glucagon-positive cell areas decreased in the STZ-treated mice fed a normal diet, with no such change in the mice fed a HFD (Fig. 3c). Additionally, Ki67 expression in beta cells was significantly increased in the NA group islets (Fig. 3e and Additional file 1: Figure S1). Therefore, the glucagon positive cell area: insulin positive cell area ratios showed regeneration only in the mice fed a normal diet (Fig. 3d).

## Discussion

Islets of Langerhans features reflect the severity and the stage of diabetes. A compensatory increase in insulin secretion due to insulin resistance is observed in the impaired-glucose-tolerance stage and in the very early stage of Type 2 diabetes mellitus (DM) [7–9]. As in Type 1 DM, in the advanced stage of Type 2 DM with hyperglycemia, the beta cell mass is usually reduced [10, 11], while that of alpha cells is unchanged or even increased [12, 13]. Thus, a drug reversing these impairments of the islets of Langerhans, achieving regeneration of beta cells, would be an ideal therapy for both forms of DM.

While DPP-4 inhibitors were initially developed to enhance glucose secretion in response to insulin, recent studies have revealed effects on not only beta cells but also alpha cells, i.e. these drugs suppress glucagon secretion [3, 14]. In addition, many studies employing in vitro techniques and experiments using rodent models have suggested DPP-4 inhibitors to suppress apoptosis as well as promoting the proliferation of beta cells [1, 15].

Herein, we clearly demonstrated that anagliptin reversed the degeneration of islets of Langerhans in STZ-treated mice, based on up-regulation of mRNA levels of the beta cell markers Pdx-1, MafA, NeuroD and Nkx6.1. On the other hand, these improvements were significantly blunted in HFD fed mice, although serum GLP-1 levels and pancreatic beta cell marker expressions were increased, to similar extents, in both normal diet and HFD fed mice. We speculate that lipotoxicity caused by the HFD may have suppressed the favorable effects exerted by anagliptin on beta cells. Yet other reports have shown that increased active GLP-1 functions in beta cells, promoting their proliferation in response to DPP-4 inhibitor administration, resulting in improved beta cell functions such as insulin secretion [2, 3, 14, 16]. Additionally, some reports have shown that insulin glargine induced phosphorylations of mitogenic factor Akt and p44/p42 MAPK in several cell lines, suggesting effects of Lantus® on beta cell proliferation [6]. Glucagon secretion from alpha cells is reportedly suppressed by GLP-1 [17] and insulin [18]. Despite the observed up-regulation of Pdx-1 expression, the pathway downstream from Pdx-1 in beta cells may be impaired by lipotoxicity [19]. In addition, recent reports have shown the development of insulin resistance in not only beta but also alpha cells via accumulated ER stress induced by saturated fatty acids, cytokines and so on [20–22].

In conclusion, our findings demonstrate the DPP-4 inhibitor anagliptin to strongly promote recovery from the STZ-induced destruction of Langerhans beta cells, but that this effect is weakened under HFD conditions. Although there are considerable differences between human diabetes and the STZ-treated mice used in this study, our results suggest the potential importance of ameliorating metabolic abnormalities, such as hyperlipidemia, for DPP-4 inhibitors to fully manifest their favorable effects on the islets of Langerhans such as promoting beta cell proliferation.

## Additional file

Additional file 1: Figure S1. Islet immunohistochemical images stained with insulin, Ki67 and Hoechst antibodies. Islet sections were co-stained with insulin and Ki67 antibodies, and then incubated with Hoechst and immune labeled with secondary antibodies. Insulin and Ki67 double-positive cells are indicated with arrows. Scale bar = 50 μm.
