# Supplementary figures and images for: High-fat diet feeding significantly attenuates anagliptin-induced regeneration of islets of Langerhans in streptozotocin-induced diabetic mice
Source: Diabetol Metab Syndr. 2015 Jun 2;7:50. doi: 10.1186/s13098-015-0047-y (PMC4475295; doi:10.1186/s13098-015-0047-y)

Supplemental Figure

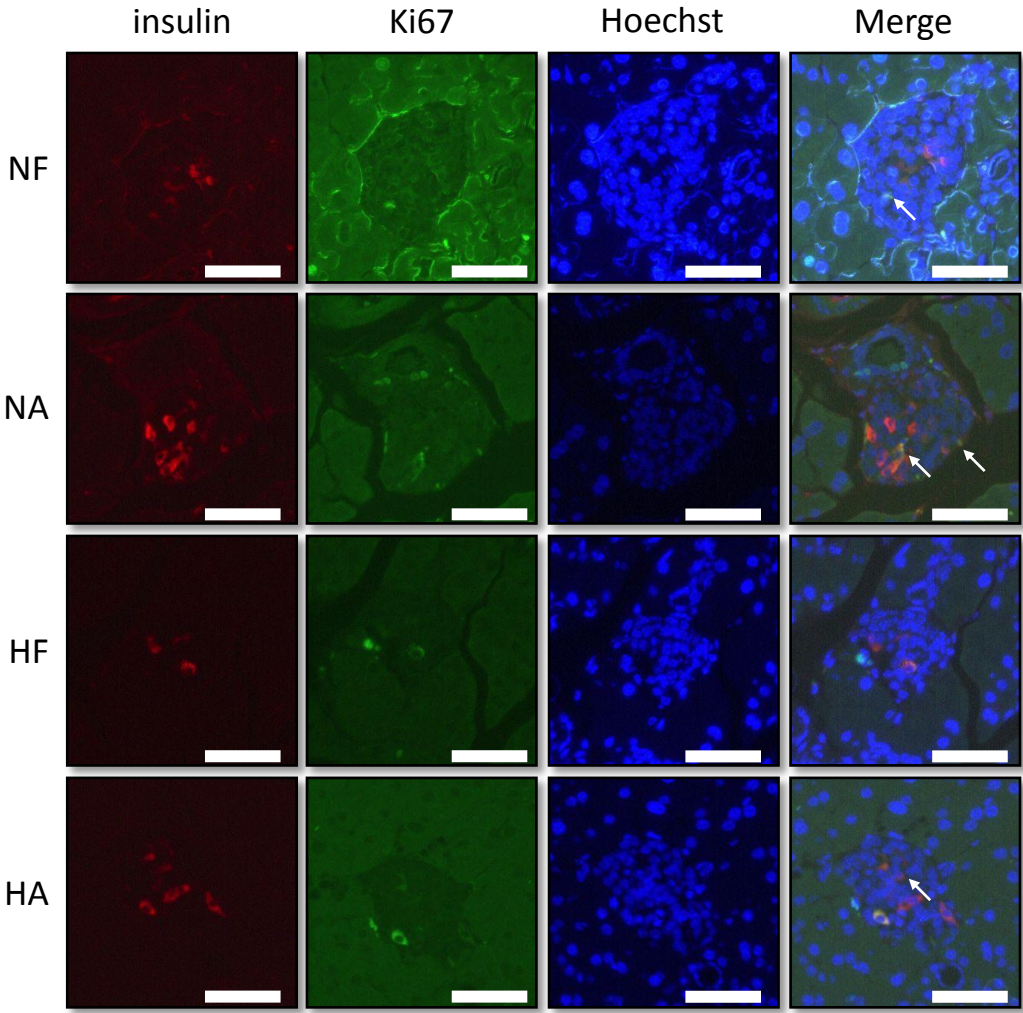

Supplement: Additional file 1: Figure S1. — Islet immunohistochemical images stained with insulin, Ki67 and Hoechst antibodies. Islet sections were co-stained with insulin and Ki67 antibodies, and then incubated with Hoechst and immune labeled with secondary antibodies. Insulin and Ki67 double-positive cells are indicated with arrows. Scale bar = 50 μm. [file 13098_2015_47_MOESM1_ESM.pdf]
